# Comparative Preclinical Evaluation of BIX-01294 and UNC0642 as EHMT2-Targeting Anticancer Agents

**DOI:** 10.3390/cancers18081250

**Published:** 2026-04-15

**Authors:** Sang Eun Park, Ji-Yoon Lee, Unju Lee, Seoyeong Kim, Nok Bi Lee, Eun Jin Cho, Seong-Yun Jeong, Soo Jin Oh, Jung Jin Hwang

**Affiliations:** 1 Asan Institute for Life Sciences, Asan Medical Center, Seoul 05505, Republic of Korea; eun6437@amc.seoul.kr (S.E.P.); a191193@amc.seoul.kr (J.-Y.L.); unjulee@amc.seoul.kr (U.L.); seoyeong@amc.seoul.kr (S.K.); syj@amc.seoul.kr (S.-Y.J.); 2 Asan Preclinical Evaluation Center for Cancer TherapeutiX, Seoul 05505, Republic of Korea; 3 Department of Medicine, University of Ulsan College of Medicine, Seoul 05505, Republic of Korea; ra02536@amc.seoul.kr (N.B.L.); cejek@amc.seoul.kr (E.J.C.)

**Keywords:** EHMT2, G9a, BIX-01294, UNC0642, pharmacokinetics, immune checkpoint blockade, anti-PD-L1

## Abstract

Background: EHMT2 (G9a) is a key epigenetic regulator frequently overexpressed in various cancers. While several inhibitors exist, their in vivo efficacy and pharmacokinetic (PK) properties remain poorly characterized. Methods: We compared the biochemical, cellular, and PK profiles of two widely used EHMT2 inhibitors, BIX-01294 and UNC0642, and evaluated their antitumor efficacy in xenograft and syngeneic mouse models. Results: Despite a higher enzymatic potency of UNC0642 (*IC*_50_ = 0.277 μM) compared to BIX-01294 (*IC*_50_ = 1.983 μM), BIX-01294 demonstrated superior cellular growth inhibition and higher intracellular accumulation. PK analysis further revealed that BIX-01294 achieved higher systemic exposure (AUC) and a wider therapeutic window via intraperitoneal administration, whereas UNC0642 exhibited dose-limiting lethality above 8 mg/kg. In HT-29 and MIA PaCa-2 xenografts, BIX-01294 (40 mg/kg) achieved up to 70.6% tumor growth inhibition (TGI), substantially surpassing UNC0642. Furthermore, in MC38 syngeneic models, a BIX-01294/anti-PD-L1 antibody combination produced an additive effect. This combination markedly increased the number of tumor-infiltrating CD8α^+^ T cells and NK1.1^+^ cells. Conclusions: These results suggest that BIX-01294 is more effective in vivo than UNC0642 due to its favorable PK profile and superior cellular uptake. Our findings support the further development of EHMT2 inhibitors as potent partners for immune checkpoint blockades.

## Simple Summary

EHMT2 (also known as G9a) is an epigenetic regulator that contributes to tumor progression and immune evasion. Although several EHMT2 inhibitors have been developed, it remains unclear which agent is most effective in vivo. In this study, we compared two EHMT2 inhibitors: BIX-01294 and UNC0642. Surprisingly, despite exhibiting weaker activity in in vitro assays, BIX-01294 more effectively suppressed tumor growth in a mice model of pancreatic and colon cancer. This enhanced efficacy was associated with greater intracellular accumulation and higher systemic drug exposure in the body, leading to more sustained target inhibition. In addition, BIX-01294 showed additive antitumor effects when combined with the anti-PD-L1 antibody, an established immunotherapy. These findings suggest that intracellular drug accumulation and pharmacokinetic properties are key determinants of therapeutic success and support the use of EHMT2 inhibitors in combination with immune checkpoint blockade for cancer treatment.

## 1. Introduction

Epigenetic modifications regulate gene expression without DNA alteration and are essential for fundamental biological processes, including development and cell cycle regulation [1]. Dysregulated epigenetic changes lead to transcriptional imbalances, which are implicated in various pathologies, particularly cancers [2]. As an example, the misregulation of histone methylation, which is maintained by the balance between histone methyltransferases (HMTs) and histone demethylases (HDMs), is frequently observed across multiple cancers [3].

Euchromatic histone-lysine N-methyltransferase 2 (EHMT2), also known as G9a, is one of the most extensively studied HMTs in oncology. G9a forms a heterodimeric complex with the closely related G9a-like protein (GLP, also known as EHMT1). This complex is responsible for the mono- and di-methylation of histone H3 Lys9 (H3K9me1/2), resulting in transcriptional silencing [4,5]. While EHMT2 expression is high during embryonic development to facilitate cell fate determination, it is significantly downregulated in most differentiated adult tissues. However, EHMT2 is frequently overexpressed in a wide range of cancers through mechanisms such as copy number gain [6], reduced degradation under hypoxic environments [7], and the loss of tumor-suppressive microRNAs, including miR-1 [8] and miR-122 [9]. Given its established role in tumorigenesis, drug resistance, and poor prognosis, EHMT2 has emerged as a compelling therapeutic target [10,11,12].

BIX-01294 was among the first selective EHMT2 inhibitors, identified via high-throughput screening in 2007 [13]. Subsequently, more potent and selective inhibitors such as UNC0638 were developed [14]. Since EHMT2 and EHMT1 share high homology in their catalytic SET and ankyrin repeat domains [15], these inhibitors typically target both enzymes. For instance, the half maximal inhibitory concentration (*IC*_50_) values of BIX-01294 and UNC0638 are 1.7 μM and 15 nM for EHMT2, respectively. Although these compounds effectively inhibit cancer cell proliferation in vitro, their utility in in vivo models has been hampered by suboptimal pharmacokinetic (PK) properties. To address this, UNC0642 was developed with improved in vivo PK profiles compared to UNC0638 [16]. Despite the discovery of several EHMT1/2 inhibitors to date, many still exhibit limited clinical drug-likeness, including off-target effects and toxicity, necessitating a more detailed investigation of their pharmacological profiles.

BIX-01294 and UNC0642 remain the most widely utilized EHMT2 inhibitors in various cancer studies [7,17,18,19]. Although these compounds have demonstrated efficacy in tumor growth inhibition (TGI) models, the reported dosages vary widely [19,20,21,22,23], and comprehensive data regarding their pharmacological properties and toxicity are lacking. In this present study, we evaluated the in vitro and in vivo PK properties, toxicity, and antitumor efficacy of BIX-01294 and UNC0642 to establish preclinical guidelines for their experimental use. Our findings facilitate future mechanistic investigations of these agents and support the development of next-generation EHMT2-targeting therapeutics.

## 2. Materials and Methods

### 2.1. Cell Culture and Reagents

Human cancer cell lines, including MIA PaCa-2, HT-29, and HCT116, were obtained from the American Type Culture Collection (Manassas, VA, USA), whereas the murine colon adenocarcinoma cell line MC38 was purchased from Kerafast Inc. (Boston, MA, USA). Cells were cultured in Dulbecco’s Modified Eagle Medium supplemented with 10% fetal bovine serum, 1% penicillin (100 U/mL)–streptomycin (100 μg/mL), and incubated at 37 °C under humidified incubator at 5% CO_2_. Human cell lines were authenticated by short tandem repeat profiling (Macrogen Inc., Seoul, Republic of Korea), and tested for mycoplasma contamination using a PCR-based detection kit (abm, Richmond, BC, Canada). BIX-01294 and UNC0642 were purchased from Sigma-Aldrich (St. Louis, MO, USA) and MedChemExpress (Monmouth Junction, NJ, USA), respectively, while unless other indicated, all other reagents were purchased from Sigma-Aldrich.

### 2.2. EHMT2 Enzyme Assay

The enzymatic activity of EHMT2 was evaluated using a LANCE^®^ Ultra G9a Histone H3 Lysine N-methyltransferase assay (PerkinElmer, Waltham, MA, USA). The reaction mixture consisted of 500 nM EHMT2 enzyme (BPS Bioscience, San Diego, CA, USA), 500 nM biotinylated histone H3 peptide (AnaSpec Inc., Fremont, CA, USA), and 30 μM S-adenosylmethionine (Sigma-Aldrich) in a buffer containing 2 mM Tris-HCl (pH 9.0), 50 mM NaCl, 1 mM DTT, and 0.01% Tween-20. Following compound incubation with BIX-01294 or UNC0642, 2 nM europium-labeled anti-H3K9me2 antibody and 50 nM ULight-streptavidin (PerkinElmer) were added. Signals were measured after a 60 min incubation, using an EnVision^®^ multimode plate reader (PerkinElmer) with excitation at 340 nm and emission at 665 nm. *IC*_50_ values were determined using GraphPad Prism software (version 9.5.1).

### 2.3. Cell Viability Assay

Cells were plated onto 96-well plates (1 × 10^3^ cells/well) and cultured overnight to allow cell attachment. Cells were then exposed to serial dilutions of the test compounds or vehicle control for 72 h prior to viability assessment using the CellTiter-Glo^®^ Luminescent Cell Viability Assay (Promega, Madison, WI, USA).

### 2.4. Western Blot Analysis

Protein samples were electrophoresed by SDS–PAGE and then transferred onto PVDF membranes. After blocking, membranes were probed with the specified primary antibodies followed by appropriate HRP-conjugated secondary antibodies, and signals were detected using western chemiluminescent substrate (Millipore, Burlington, MA, USA).

### 2.5. Xenograft and Syngeneic Animal Models

All animal procedures were performed under the approval of the Institutional Animal Care and Use Committee (IACUC) of Asan Medical Center. For xenograft studies, 6-week-old female BALB/c nude mice (JA BIO Inc., Seoul, Republic of Korea) were subcutaneously implanted in the right flank with 4 × 10^6^ MIA PaCa-2 or HT-29 cells. When tumors reached approximately 100 mm^3^, mice received intraperitoneal administration of BIX-01294 or UNC0642 at the indicated doses five times per week for three weeks. The compounds were prepared in a vehicle consisting of dimethylacetamide, Tween 80, and 20% (2-hydroxypropyl)-β-cyclodextrin (1:1:8, *v*/*v*). For the syngeneic model, 6-week-old female C57BL/6 mice were injected subcutaneously with 1 × 10^5^ MC38 cells. Upon tumor establishment (approximately 50–100 mm^3^), animals were randomly assigned to treatment groups, including isotype control (IgG2b, 10 mg/kg, i.p.), BIX-01294 (30 mg/kg, i.p., five times per week), anti-PD-L1 antibody (clone 10F.9G2, Bio X Cell; 10 mg/kg, i.p., administered every 3 days for a total of four doses), or combination treatment. Tumor growth was assessed twice per week by measuring dimensions with a digital caliper, and tumor volume was calculated according to the formula: (length × width^2^)/2.

### 2.6. Immunocytochemistry

Tumor tissues obtained from the MC38 model were embedded in Optimal Cutting Temperature (OCT) compound (Tissue-Tek^®^, Torrance, CA, USA) and cryosectioned. Tissue sections were fixed with 4% paraformaldehyde, incubated with primary antibodies against CD8α (Abcam, Cambridge, UK) and NK1.1 (CD161; Novus Biologicals, Centennial, CO, USA) at 4 °C. After washing, sections were incubated with Alexa Fluor^®^ 488-conjugated secondary antibodies (Thermo Fisher Scientific, Waltham, MA, USA) for 1 h. Slides were mounted in VectaShield^®^ medium (Vector Laboratories, Newark, CA, USA) following nuclear counterstaining with DAPI (Sigma-Aldrich). Fluorescence images were captured using an EVOS FL Auto2 imaging system (Thermo Fisher Scientific), and quantitative analysis was performed using Fiji software (National Institutes of Health, Bethesda, MD, USA).

### 2.7. In Vivo Pharmacokinetic Studies

In vivo exposure profiles of the BIX-01294 and UNC0642 were evaluated in male ICR mice (8 weeks old; Orient-Bio, Seongnam, Republic of Korea). To assess route-dependent differences in systemic exposure, compounds were administered via intravenous (i.v.), oral (p.o), or intraperitoneal (i.p.) routes at dose levels of 1 or 5 mg/kg, as appropriate. Serial blood samples were obtained through the saphenous vein at predefined time points throughout the study period. Whole blood samples were processed to ensure efficient recovery of analytes from whole blood. Briefly, cellular lysis was achieved repeated freeze-thaw cycles, followed by protein precipitation using an organic solvent containing carbamazepine as an internal standard. Samples were then vigorously vortex-mixed and subjected to ultrasonic agitation to enhance extraction efficiency. Following centrifugation at 4 °C, the clarified supernatant was collected for analysis. Quantitative determination of analytes was performed using tandem mass spectrometry coupled to liquid chromatography (LC-MS/MS). Chromatographic separation was achieved on a reversed-phase column using a gradient elution program with acidified aqueous and organic mobile phases. Detection was carried out on a triple quadrupole mass spectrometer (API 4000, Sciex, Foster City, CA, USA) coupled to an HPLC system (Agilent 1200 series, Agilent, Santa Clara, CA, USA), operating in multiple reaction monitoring (MRM) mode with compound-specific ion transitions to ensure analytical selectivity and sensitivity. Pharmacokinetic parameters describing systemic exposure were obtained using a non-compartmental analysis (NCA) implemented in Kinetica™ software (version 4.4.1).

### 2.8. Permeability and Stability Assay

To evaluate intestinal permeability, Caco-2 cells were cultured on Transwell^®^ permeable inserts for 20–25 days to allow differentiation into confluent monolayers exhibiting functional tight junctions, as previously described [24]. The integrity of the monolayer was confirmed prior to the assay. Test compounds were applied to the apical side (donor) in buffered solution (HBSS, pH 7.4), and their transport to the basolateral (receiver) compartment was monitored over a 2 h incubation period under controlled conditions. Samples were collected at designated time points, and transport efficiency was quantified as the apparent permeability coefficient (P*_app_*), calculated from the rate of compound appearance in the receiver compartment normalized to the initial donor concentration.

To assess metabolic susceptibility, in vitro incubations were conducted using pooled liver microsomal preparations of mouse and human origin. Enzymatic reactions were initiated by the addition of an NADPH and carried out at 37 °C. At predefined time points, aliquots were withdrawn and immediately quenched with ice-cold organic solvent to terminate enzymatic activity. The concentration of the parent compound remaining at each time point was determined by liquid chromatography–tandem mass spectrometry (LC–MS/MS). All analytical data were acquired and processed using Analyst™ software (version 1.6.2).

### 2.9. Statistical Analysis

Data are expressed as mean values ± SD as indicated. Statistical significance was determined using a two-tailed Student’s *t*-test, or one-way or two-way ANOVA, followed by multiple comparison corrections. *p* values < 0.05 was considered statistically significant. All experiments were performed in triplicate.

## 3. Results

### 3.1. BIX-01294 Exhibits Superior Cellular Antitumor Efficacy Despite Lower Enzymatic Potency

**Figure 1:**
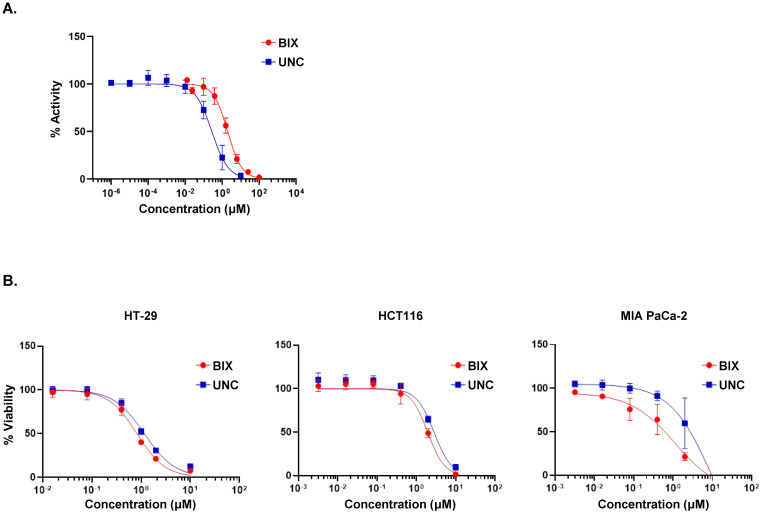
In vitro effects of the EHMT2 inhibitors BIX-01294 and UNC0642 on enzymatic activity, cell viability, and histone methylation. (**A**) Inhibition of EHMT2 enzymatic activity by BIX-01294 and UNC0642 measured using a LANCE^®^ Ultra G9a Histone H3 Lysine N-methyltransferase assay. Data are presented as normalized fluorescence signals (%) versus compound concentration. (**B**) Cell viability following 72 h treatments with BIX-01294 or UNC0642 in HT-29, HCT116, and MIA PaCa-2 cells. Viability was measured using a CellTiter-Glo^®^ luminescence-based assay and expressed as a percentage relative to vehicle control. BIX, BIX-01294; UNC, UNC0642.

**Figure 2:**
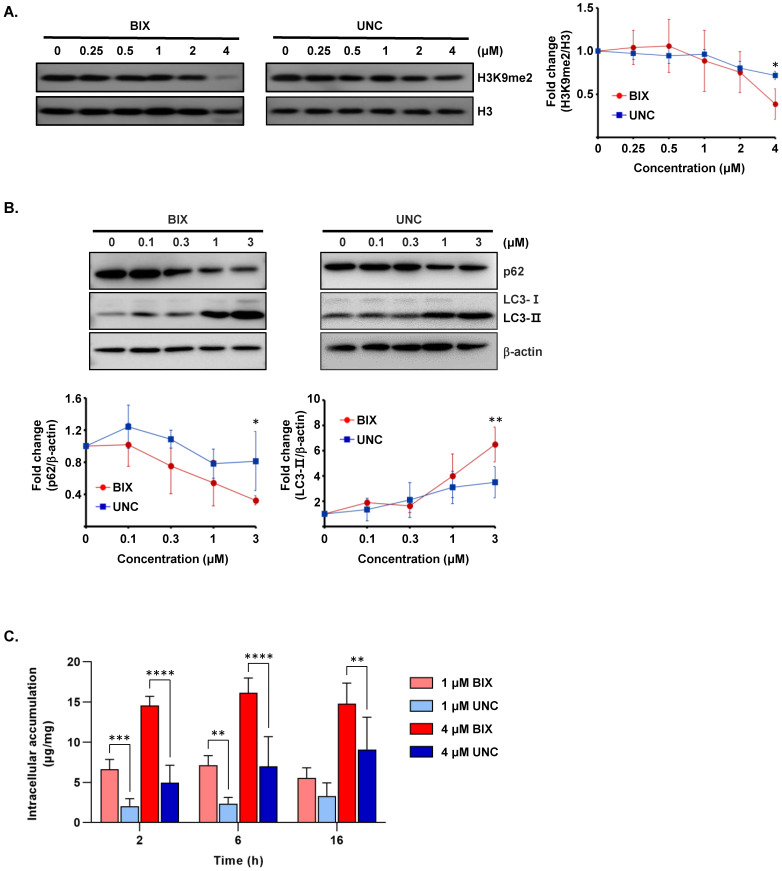
Comparative effects of BIX-01294 and UNC0642 on epigenetic regulation and autophagic signaling in MIA PaCa-2 cells. (**A**) Western blot analysis of H3K9me2 levels in MIA PaCa-2 cells treated with increasing concentrations (0–4 μM) of BIX-01294 or UNC0642 for 4 days. Total histone H3 served as a loading control. Densitometric quantification of H3K9me2 relative to H3 is shown on the right panel. (**B**) Western blot analysis of autophagy-related markers p62 and LC3-II in MIA PaCa-2 cells treated with BIX-01294 or UNC0642 (0–3 μM) for 72 h. β-actin was used as a loading control. Densitometric quantifications normalized to β-actin are shown on the lower panel. (**C**) Intracellular accumulation of BIX-01294 and UNC0642 in MIA PaCa-2 cells. MIA PaCa-2 cells were treated with BIX-01294 or UNC0642 at concentrations of 1 μM and 4 μM. At 2, 6, and 16 h post-treatment, the intracellular compound levels were measured and normalized to the total protein content (μg/mg). Data are presented as a mean ± SD. * *p* < 0.05, ** *p* < 0.01, *** *p* < 0.005, and **** *p* < 0.001 versus corresponding UNC-treated groups. BIX, BIX-01294; UNC, UNC0642; h, hour. The original Western blot figures can be found in Supplementary File S1.

To assess the inhibitory potency of the EHMT2 inhibitors, we first measured their enzymatic activity. As expected, UNC0642 demonstrated a markedly stronger inhibition of EHMT2 enzymatic activity, with an *IC*_50_ value of 0.277 ± 0.21 μM, compared to BIX-01294, which exhibited an *IC*_50_ of 1.983 ± 0.47 μM (Table 1 and Figure 1A). UNC0642 thus demonstrated more potency as an EHMT2 inhibitor in biochemical assays. It was notable however that cellular assays revealed contrasting results to these in vitro tests. In cell viability assays across HT-29, HCT116, and MIA PaCa-2 cell lines at 72 h, BIX-01294 consistently exhibited enhanced antiproliferative effects (Figure 1B), showing lower *IC*_50_ values (0.822 μM to 1.945 μM) than UNC0642 (1.660 μM to 2.822 μM) across all tested cell lines (Table 1). To further investigate these findings, we analyzed the dimethylation of histone H3 Lys9 (H3K9me2) in MIA PaCa-2 cells following a 4-day treatment to allow for gradual epigenetic changes. Both compounds reduced H3K9me2 levels in a concentration-dependent manner, but BIX-01294 induced a more robust suppression than UNC0642 (Figure 2A).

Since EHMT2 inhibition is associated with autophagy induction [25,26,27], we further examined autophagy markers in the presence of the inhibitors. Both agents decreased the p62 levels and increased the LC3-II levels in a concentration-dependent manner, with more pronounced changes observed in BIX-01294-treated cells (Figure 2B). To investigate the underlying basis for this superior cellular potency of BIX-01294, we quantified the intracellular accumulation of both compounds in MIA PaCa-2 cells. At a concentration of 4 μM, BIX-01294 showed three-fold-higher intracellular accumulation than UNC0642 at 2 h, maintaining a two-fold difference up to 16 h. Similarly, at 1 μM, BIX-01294 levels were initially four-fold higher and remained approximately two-fold greater than UNC0642 by 16 h (Figure 2C). These findings suggested that BIX-01294 is more efficiently taken up or retained within cells, accounting for its stronger cellular effects despite its lower biochemical potency.

### 3.2. Pharmacokinetic Profiles Support the Superior In Vivo Efficacy of BIX-01294

**Figure 3:**
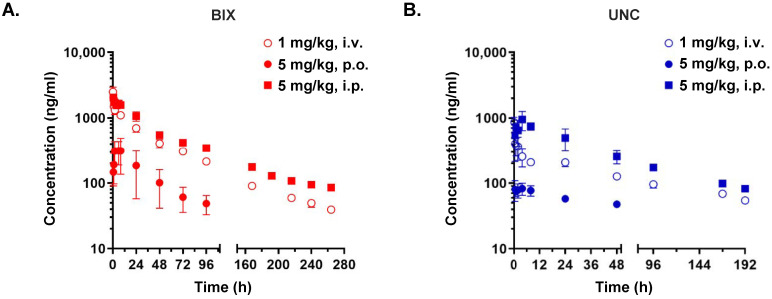
Blood concentration–time profiles of BIX-01294 and UNC0642 in male ICR mice. (**A**,**B**) A single dose of BIX-01294 (**A**) or UNC0642 (**B**) was administered intravenously (○; 1 mg/kg), orally (●; 5 mg/kg), or intraperitoneally (■; 5 mg/kg). Blood concentrations were monitored up to 264 h post-dosing. Data are presented as a mean ± SD. BIX, BIX-01294; UNC, UNC0642; i.v., intravenous; p.o., oral; i.p., intraperitoneal; h, hour.

To characterize their PK profiles, blood concentration–time curves were generated following i.v., p.o., and i.p. administration of BIX-10294 and UNC0642 in male ICR mice. PK parameters were calculated using non-compartmental analysis. Following the i.v. administration of BIX-01294 (1 mg/kg), blood concentrations remained above the lower limit of quantification (LLOQ) for up to 264 h (Figure 3A). BIX-01294 exhibited high systemic exposure, with an area under the curve (AUC) of 69,949.9 ± 6131.1 ng·h/mL (Table 2). This compound further showed low systemic clearance (CL = 0.01 L/h/kg) and a large volume of distribution at steady state (*V_ss_* = 1.08 ± 0.12 L/kg), consistent with a slow systemic elimination. Correspondingly, the mean residence time (MRT) and terminal half-life (*t*_1/2_) of BIX-01294 were 79.78 h and 80.12 h, respectively. Collectively, these parameters suggested an extensive tissue distribution and/or strong plasma protein binding, contributing to the prolonged systemic persistence of BIX-01294.

After i.v. administration of UNC0642 (1 mg/kg), quantifiable blood concentrations were observed for up to 192 h (Figure 3B). This inhibitor showed relatively low systemic exposure with an AUC of 22,546.9 ± 734.1 ng·h/mL, despite displaying a similarly low clearance (CL = 0.03 L/h/kg) and a markedly larger volume of distribution (*V_ss_* = 5.06 ± 0.57 L/kg) (Table 2). The MRT and *t*_1/2_ were 166.27 h and 128.33 h, respectively, indicating prolonged systemic retention. Nevertheless, the overall systemic exposure of UNC0642 remained notably lower than that of BIX-01294.

Following oral administration at 5 mg/kg, both compounds exhibited poor oral bioavailability (*F*), with values of 3.7% for BIX-01294 and 2.6% for UNC0642. Pharmacokinetic parameters, which require terminal elimination rate constant on their calculation, could not be determined following the p.o. dose, as the terminal elimination phase could not be accurately defined. This low oral exposure likely results from limited gastrointestinal absorption, poor aqueous solubility, and/or first-pass metabolism. These findings were further supported by Caco-2 permeability assays, where both BIX-01294 and UNC0642 showed relatively low apparent permeability coefficients (P*_app_*) of 1.4 × 10^−6^ cm/s and 1.7 × 10^−6^ cm/s, respectively (Table 3). These values were comparable to, or lower than, those of low permeability references atenolol or ranitidine.

In contrast to the oral dose findings, i.p. administration markedly enhanced the systemic exposure of the two EHMT2 inhibitors. For BIX-01294 (5 mg/kg), the MRT was 99.56 h and the AUC was 104,361.9 ± 6840.6 ng·h/mL. The *F* value was 29.84%, which was approximately eight-fold higher than that observed following oral administration (Table 2). UNC0642 also showed improved exposure after i.p. dosing, with an MRT of 107.66 h, an AUC of 47,413.3 ± 8470.0 ng·h/mL and an *F* value of 42.06%. Notably, although i.p. administration improved the bioavailability of both compounds, BIX-01294 achieved a total systemic exposure of more than twice that of UNC0642.

**Figure 4:**
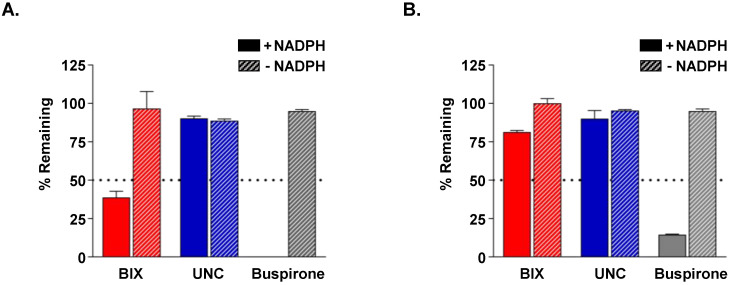
In vitro metabolic stability of BIX-01294 and UNC0642. (**A**,**B**) Metabolic stability of 1 μM BIX-01294, UNC0642, and buspirone (a positive control) measured in mouse (**A**) or human (**B**) liver microsomes in the absence (hatched bar) or presence (filled bar) of 1 mM NADPH for 30 min at 37 °C. Data are expressed as a mean ± SD. BIX, BIX-01294; UNC, UNC0642; NADPH, nicotinamide adenine dinucleotide phosphate.

To assess their susceptibility to cytochrome P450 (CYP)-mediated oxidative metabolism, BIX-01294 and UNC0642 were incubated with pooled mouse or human liver microsomes in the presence of NADPH. After incubation for 30 min, 38.6% (mouse) and 81.3% (human) of BIX-01294 and 90.2% (mouse) and 90.1% (human) of UNC0642 remained (Figure 4). Although BIX-01294 exhibited more rapid metabolism in mouse liver microsomes than UNC0642, its substantially higher AUC and prolonged half-life following i.p. injection indicated that its extensive tissue distribution and high systemic exposure outweighed its susceptibility to oxidative metabolism in mice.

Taken together, these PK findings support the selection of the i.p. route for subsequent in vivo studies and suggest that BIX-01294 will demonstrate superior therapeutic activity compared with UNC0642 as an EHMT2 inhibitor due to its relatively higher systemic exposure.

### 3.3. BIX-01294 Suppresses Tumor Growth and Enhances Antitumor Immunity In Vivo

We next evaluated the in vivo antitumor efficacy of both compounds in xenograft models to confirm if the superior cellular effects of BIX-01294 could be recapitulated. In preliminary dose-finding toxicity tests, UNC0642 showed acute toxicity and lethality at 10 mg/kg (i.p.), leading us to set the maximum dose at 8 mg/kg. Conversely, BIX-01294 was well-tolerated at 40 mg/kg, though 80 mg/kg proved lethal. Accordingly, mice bearing HT-29 or MIA PaCa-2 tumors were treated with these maximum established doses (i.p.) for three weeks.

**Figure 5:**
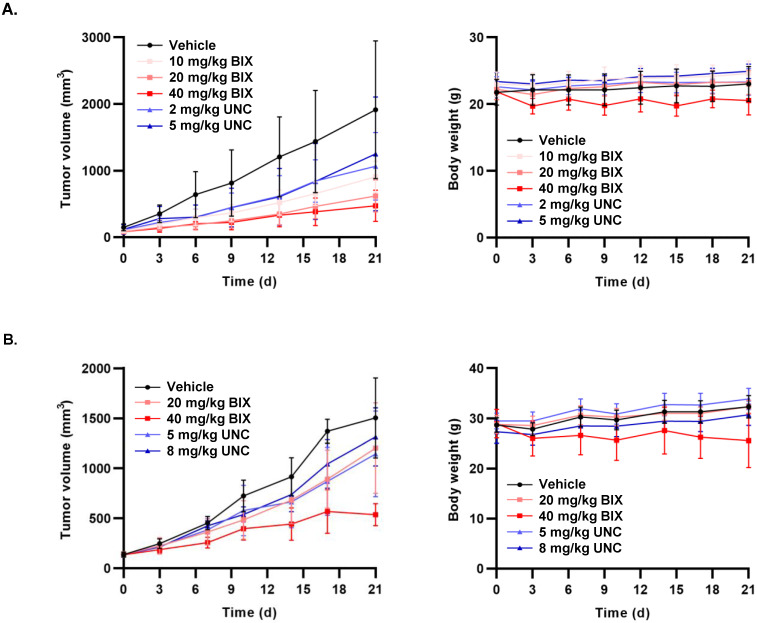
In vivo antitumor efficacy of EHMT2 inhibitors in xenograft models. (**A**) Tumor growth (**left**) and body weight monitoring (**right**) in HT-29 tumor-bearing mice treated with BIX-01294 (10, 20, or 40 mg/kg) or UNC0642 (2 or 5 mg/kg) for 24 days. (*n* = 5–6 per group). (**B**) Tumor growth (**left**) and body weight (**right**) in MIA PaCa-2 tumor-bearing mice treated with BIX-01294 (20 or 40 mg/kg) or UNC0642 (5 or 8 mg/kg) via intraperitoneal injection for 21 days. Data are presented as a mean ± SD. BIX, BIX-01294; UNC, UNC0642; d, Day.

In our HT-29 xenograft mouse models, the administration of BIX-01294 at 10, 20, or 40 mg/kg resulted in a 19.82%, 45.45%, or 58.44% reduction in tumor size, respectively (Figure 5A). In contrast, UNC0642 treatments (2 or 5 mg/kg, i.p.) inhibited tumor growth less effectively than BIX-01294 and failed to exhibit a clear dose-dependent response (Figure 5A). Consistent with these findings, the same experiments in an MIA PaCa-2 xenograft model also indicated a superior antitumor activity of BIX-01294 over UNC0642. Treatment with BIX-01294 at 20 and 40 mg/kg, i.p. led to dose-dependent TGI rates of 22.1% and 70.6%, respectively, whereas UNC0642 at 5 and 8 mg/kg i.p. reduced tumor size by only 26.0% and 13.8% (Figure 5B). Regarding safety, although 40 mg/kg BIX-01294 induced a modest reduction in body weight (11.8%), no significant weight changes were observed in any other treatment groups, including UNC0642 (Figure 5A,B).

**Figure 6:**
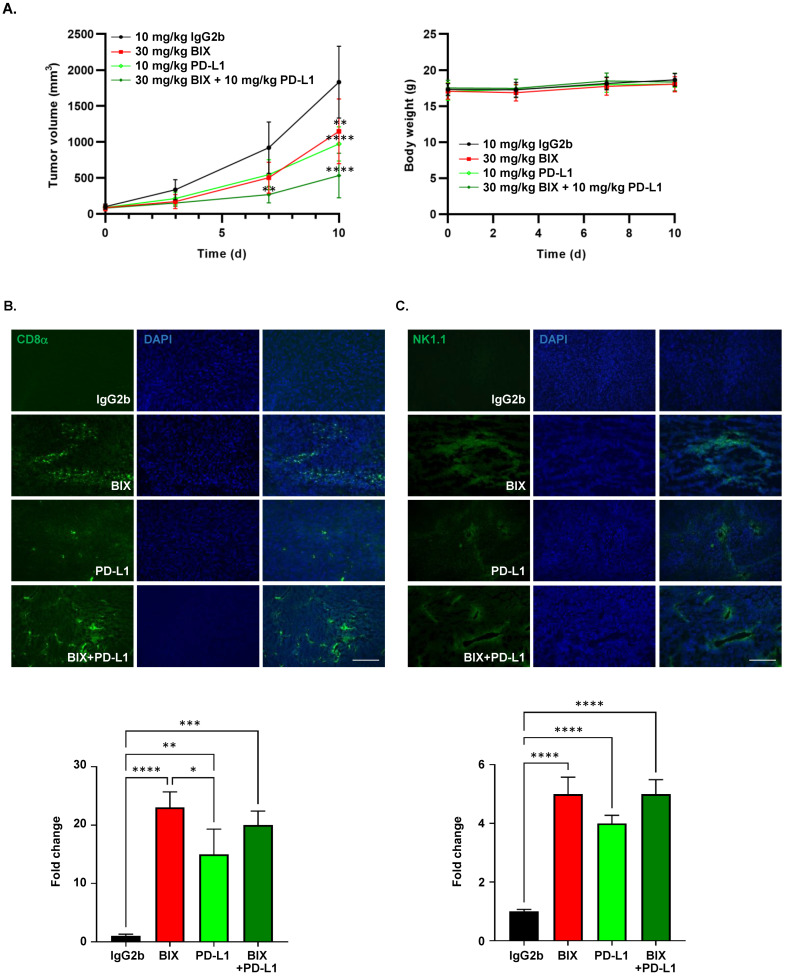
Antitumor activity of BIX-01294 in combination with a PD-L1 blockade in an MC38 syngeneic tumor model. (**A**) Tumor growth (**left**) and body weight changes (**right**) in C57BL/6 mice bearing MC38 tumors treated with control IgG2b (10 mg/kg), BIX-01294 (30 mg/kg), anti-PD-L1 antibody (10 mg/kg), or a combination (*n* = 5 per group). (**B**,**C**) Immunofluorescence analysis of tumor-infiltrating CD8α^+^ T cells (**B**) and NK1.1^+^ cells (**C**) in MC38 tumors. DAPI was used for nuclear counterstaining. Quantifications of fluorescence intensity per tumor area are shown below each image. Scale bar, 125 µm. Data are expressed as a mean ± SD. * *p* < 0.05 versus BIX and ** *p* < 0.01, *** *p* < 0.005, and **** *p* < 0.001 versus vehicle group. BIX, BIX-01294; PD-L1, anti-PD-L1 antibody; d, Day.

Building upon the superior in vivo antitumor activity of BIX-01294 in the xenograft models, we investigated its potential for enhanced therapeutic efficacy in combination with an immune checkpoint blockade. We investigated the effects of BIX-01294 in combination with an anti-PD-L1 antibody in an MC38 syngeneic colon cancer model. The combination treatment resulted in markedly enhanced tumor suppression (72.0%) compared with BIX-01294 (34.6%) or anti-PD-L1 antibody (45.6%) monotherapies (Figure 6A). Importantly, this enhanced efficacy did not induce significant body weight loss. Immunofluorescence staining demonstrated a marked increase in intratumoral CD8α^+^ cytotoxic T cells and NK1.1^+^ natural killer (NK) cells following treatment with BIX-01294 alone, which was further sustained in the combination group with anti-PD-L1 antibody (Figure 6B,C). These findings indicate that BIX-01294 contributes to antitumor immunity by promoting T and NK cell infiltration, and that this effect is maintained or modestly enhanced in combination with a PD-L1 blockade.

## 4. Discussion

The development of EHMT2 inhibitors has attracted considerable attention due to the pivotal role of EHMT2 in promoting tumor progression and modulating the tumor microenvironment [4,5]. Our present findings, however, have revealed a compelling divergence between biochemical potency and therapeutic outcomes: While UNC0642 exhibits superior biochemical potency against the EHMT2 enzyme, BIX-01294 demonstrates markedly greater cellular uptake, systemic exposure, and both superior in vitro and in vivo antitumor efficacy. These discrepancies highlight the critical necessity of evaluating intracellular accumulation alongside enzymatic inhibition during preclinical development. Our current data suggest that the robust in vivo performance of BIX-01294 is primarily driven by its superior PK profile and wider therapeutic window, which facilitated efficacious exposure levels that were unattainable with UNC0642 due to its acute toxicity.

An important finding of our present study is that enzymatic *IC*_50_ values are not always predictive of cellular or in vivo outcomes. This phenomenon was consistently observed across multiple cancer cell lines, indicating that the superior cellular efficacy of BIX-01294 is not cell line-specific. This effect is likely driven by enhanced intracellular accumulation, as observed particularly in MIA PaCa-2 cells. In addition, cell-type-dependent factors such as drug transporter activity (e.g., efflux pumps) and resistance-related mechanisms may further influence intracellular retention and cellular response. Although UNC0642 was approximately 7-fold more potent than BIX-01294 in cell-free enzymatic assays, BIX-01294 achieved consistently lower *IC*_50_ values in HT-29, HCT116, and MIA PaCa-2 cell lines. Our intracellular accumulation data, showing a four-fold-higher accumulation of BIX-01294 than UNC0642 in MIA PaCa-2 cells (Figure 2C), provided a mechanistic explanation for this inconsistency. This enhanced cellular retention likely facilitates a superior suppression of H3K9me2 and the more pronounced changes in the autophagic markers, p62 and LC3-II. These results have indicated that measuring intracellular drug concentrations can significantly aid in the interpretation of in vitro efficacy. While we established a link between the high cellular uptake of BIX-01294 and its efficacy, the specific structural features and transporters responsible for this superior permeability remain to be elucidated.

Our current PK analyses further elucidated the differential antitumor activity observed between BIX-01294 and UNC0642 in animal models. While BIX-01294 is often characterized as having suboptimal PK profiles [28], our present analyses indicated that i.p. administration of this compound yields a systemic exposure (AUC) of more than two-fold higher than that of UNC0642. This higher exposure, coupled with extensive tissue distribution and high intracellular accumulation, appears to compensate for the relatively higher metabolic susceptibility of BIX-01294 in mouse liver microsomes. Although it was generally considered a favorable pharmacokinetic profile, characterized by increased AUC and extended half-life, systemic exposure metrics alone are insufficient to predict antitumor efficacy. Semi-mechanistic tumor growth inhibition models, such as the Simeoni model, are widely used to assess whether AUC, C_max_, or C_trough_ best correlates with therapeutic response [29,30]. For epigenetic targets such as EHMT2, sustained target engagement is critical, suggesting that maintaining drug concentrations above an effective threshold (C_trough_) may be more relevant than achieving high peak levels (C_max_), consistent with the importance of drug-target residence time [31,32]. While UNC0642 exhibits a high volume of distribution, the unbound fraction (F_u_) at the site of action remains unknown. Therefore, integrated PK/PD modeling that links systemic exposure, in vitro potency (*IC*_50_), and tumor growth inhibition will be essential to define the primary determinants of in vivo efficacy.

The therapeutic utility of these inhibitors was further dictated by their respective safety margins and dose-limiting toxicities. In our dose-escalation studies, we established the maximal tolerated doses (MTDs) and observed broader therapeutic window for BIX-01294. Specifically, BIX-01294 could be safely administered intraperitoneally up to 40 mg/kg at dosages required for robust tumor inhibition within a manageable and reversible toxicity range. Whereas UNC0642 was strictly limited by acute toxicity at relatively low doses. Intraperitoneal doses exceeding 8 mg/kg of UNC0642 resulted in acute lethality, preventing the attainment of exposure levels of UNC0642 required for robust tumor inhibition. Since our toxicity assessments were conducted in murine models however, the acute lethality observed with UNC0642 must be interpreted carefully before translating these safety profiles to the human context. When both compounds were administered at dose levels below the MTD over a 3-week repetitive dosing period, histopathological analysis of H&E-stained organ sections, including liver, kidney, stomach, intestine, and heart, revealed no significant pathological events for either drug. Nonetheless, for future clinical translations, these findings have emphasized that pharmacokinetic exposure and safety margins are more critical points than simple biochemical potency.

In addition to its direct antitumor effects, our present study has highlighted a potent synergy between BIX-01294 and anti-PD-L1 therapy. While BIX-01294 monotherapy showed moderate effects, its combination with PD-L1 blockade led to a dramatic 72% reduction in tumor growth. It has been reported that oncogenic activation or overexpression of EHMT2 correlates with a cold immune microenvironment and poor response to immune checkpoint inhibitors [33]. Consistently, EHMT2 inhibition was found to enhance the immune checkpoint blockade response via the activation of pro-inflammatory pathways and triggering of immunogenic cell death [34,35]. Our current immunofluorescence data (Figure 6B,C) further support the hypothesis that BIX-01294 warms up the tumor microenvironment, as evidenced by the significantly increased infiltration of CD8α^+^ cytotoxic T cells and NK1.1^+^ natural killer cells. While the combination treatment showed a clear additive effect on tumor volume reduction (Figure 6A), the increase in tumor-infiltrating CD8+ and NK1.1+ cells in the combination group was not significantly higher than that observed with BIX-01294 monotherapy (Figure 6B,C). In this MC38 model, BIX-01294 monotherapy may induce a near-maximal level of immune cell infiltration, which is then maintained in the combination group. This suggests that BIX-01294 not only inhibits tumor cell proliferation directly but also reprograms the tumor microenvironment to be more receptive to T-cell- and NK-cell-mediated immunosurveillance. Although we observed increased infiltration by immune cells, further mechanistic studies are needed for the continued refinement of EHMT2-targeting therapeutics.

Our present study findings have significant implications for the clinical development of next-generation EHMT2 inhibitors as they indicate that the lead optimization process must simultaneously address cellular permeability and systemic exposure alongside biochemical potency for successful drug development. The failure of UNC0642 to achieve efficacy due to its narrow therapeutic index serves as a cautionary example of this. Further drug discovery efforts should prioritize scaffolds that offer a broader safety margin and superior tissue distribution, even if they exhibit relatively modest enzymatic *IC*_50_ values. Moreover, the robust synergy we here observed in combination with immune checkpoint blockade continues to position EHMT2 inhibitors as promising candidates for combinatorial strategies in patients who are non-responsive to current immunotherapies.

## 5. Conclusions

In conclusion, our study demonstrates that intracellular accumulation and pharmacokinetic properties are key determinants of the therapeutic efficacy of EHMT2 inhibitors. Despite its lower enzymatic potency, BIX-01294 exhibited superior cellular uptake, systemic exposure, and antitumor activity compared to UNC0642. Furthermore, BIX-01294 significantly enhanced the efficacy of the immune checkpoint blockade, suggesting its potential as a combinatorial therapeutic agent. These findings underscore the importance of integrating pharmacokinetic and intracellular distribution parameters into the drug development process and provide a rationale for the continued development of EHMT2 inhibitors with optimized bioavailability and safety profiles.

## Figures and Tables

**Table 1:** *IC*_50_ values of BIX-01294 and UNC0642 for enzyme and cell viability.

Compound	Enzyme *IC*_50_ (μM)	Cell Viability *IC*_50_ (μM)		
		HT-29	HCT116	MIA PaCa-2
BIX-01294	1.983 ± 0.47	0.822 ± 0.24	1.945 ± 0.30	0.700 ± 0.59
UNC0642	0.277 ± 0.21	1.660 ± 0.26	2.822 ± 0.01	2.560 ± 1.37

**Table 2:** Pharmacokinetic profiles of BIX-01294 and UNC0642 following intravenous or intraperitoneal administration in male ICR mice.

PK Parameter		BIX-01294		UNC0642	
		IV	IP	IV	IP
		Mean ± SD	Mean ± SD	Mean ± SD	Mean ± SD
Dose	(mg/kg)	1	5	1	5
t_max_	(h)	NA	0.50 ± 0.00	NA	3.00 ± 1.73
C_max_	(ng/mL)	NA	2053.3 ± 148.4	NA	1028.33 ± 183.05
AUC_last_	(ng.h/mL)	69,949.9 ± 6131.1	104,361.9 ± 6840.6	22,546.9 ± 734.1	47,413.3 ± 8470.0
AUC_inf_	(ng.h/mL)	74,509.2 ± 6528.1	115,662.0 ± 7872.7	32,752.2 ± 2050.1	58,106.3 ± 8018.9
CL	(L/h/kg)	0.01 ± 0.00	NA	0.03 ± 0.00	NA
V_ss_	(L/kg)	1.08 ± 0.12	NA	5.06 ± 0.57	NA
V_z_	(L/kg)	1.56 ± 0.08	NA	5.63 ± 0.76	NA
t_1/2_	(h)	80.12 ± 4.73	91.78 ± 1.99	128.33 ± 23.61	90.11 ± 7.70
MRT_inf_	(h)	79.78 ± 2.14	99.56 ± 0.37	166.27 ± 27.69	107.66 ± 13.05
F	(%)	NA	29.84 ± 1.96	NA	42.06 ± 7.51

Pharmacokinetic parameters were calculated by non-compartmental analysis of the plasma concentration–time curves obtained following a single dose of BIX-01294 or UNC0642 (1 mg/kg for intravenous and 5 mg/kg for intraperitoneal dosing) in male ICR mice. Values are a mean ± S.D. (*n* = 3). NA, not applicable.

**Table 3:** Permeability of BIX-01294 and UNC0642 in Caco-2 cell.

Compound	*P_app_* × 10^−6^ (cm/s)
BIX-01294	1.4 ± 0.4
UNC0642	1.7 ± 0.2
Metoprolol	19.8 ± 1.9
Atenolol	1.3 ± 0.2
Ranitidine	2.4 ± 0.7

P*_app_* values were determined in the AP-to-BL direction. Values are a mean ± S.D. (*n* = 3). Metoprolol, atenolol, and ranitidine were included as reference compounds representing high and low permeability.

## Data Availability

The data presented in this study are available from the corresponding authors upon reasonable request.

## Supplementary Materials

The following supporting information can be downloaded at: https://www.mdpi.com/article/10.3390/cancers18081250/s1, File S1: The original Western blot figures.

## Abbreviations

The following abbreviations are used in this manuscript:

AUC	Area under the curve
CL	Clearance
CYP	Cytochrome P450
EHMT2	Euchromatic histone-lysine N-methyltransferase 2
GLP	G9a-like protein
H3K9me1/2	Mono- and di-methylation of histone H3 Lys9
HDMs	Histone demethylases
HMTs	Histone methyltransferases
i.p.	Intraperitoneal
IS	Internal standard
i.v.	Intravenous
LLOQ	Lower limit of quantification
MRT	Mean residence time
NK	Natural killer
PK	Pharmacokinetic
p.o.	Oral
TGI	Tumor growth inhibition
