# Supplementary figures and images for: Comparative Preclinical Evaluation of BIX-01294 and UNC0642 as EHMT2-Targeting Anticancer Agents
Source: Cancers (Basel). 2026 Apr 15;18(8):1250. doi: 10.3390/cancers18081250 (PMC13115232; doi:10.3390/cancers18081250)

Figure 2A

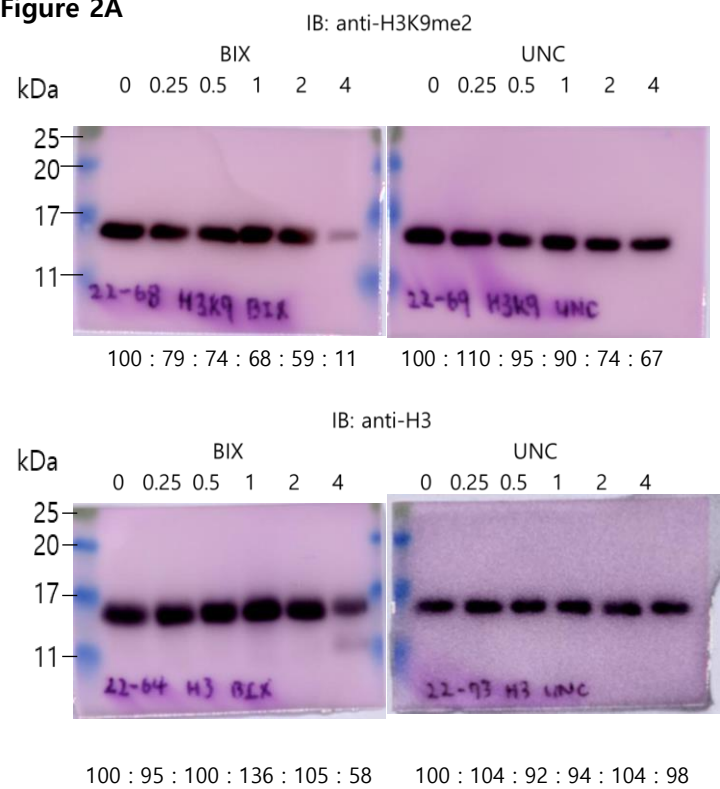

H3K9me2/H3 Ratio

1.0 : 0.8 : 0.7 : 0.5 : 0.6 : 0.2                      1.0 : 1.1 : 1.0 : 1.0 : 0.7 : 0.7

**Figure 2B**

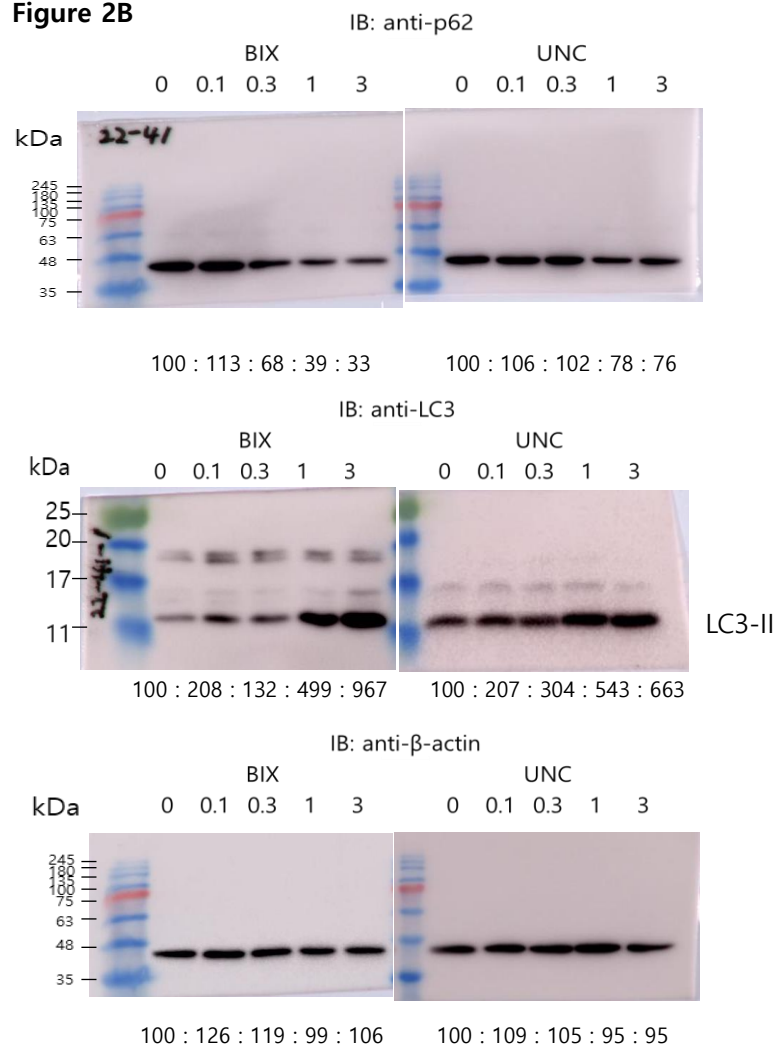

**p62/β-actin Ratio**

1.0 : 0.9 : 0.6 : 0.4 : 0.3                      1.0 : 1.0 : 1.0 : 0.8 : 0.8

**LC3-II/β-actin Ratio**

1.0 : 1.7 : 1.1 : 5.1 : 9.1                      1.0 : 1.9 : 2.9 : 5.7 : 7.0

Supplement: Supplementary file 1 [file cancers-18-01250-s001.zip › Supplementary File S1. The original Western blot figures.pdf]
